# Griseofulvin stabilizes microtubule dynamics, activates p53 and inhibits the proliferation of MCF-7 cells synergistically with vinblastine

**DOI:** 10.1186/1471-2407-10-213

**Published:** 2010-05-19

**Authors:** Krishnan Rathinasamy, Bhavya Jindal, Jayant Asthana, Parminder Singh, Petety V Balaji, Dulal Panda

**Affiliations:** 1 Department of Biosciences and Bioengineering, Indian Institute of Technology Bombay, Mumbai - 400076, Maharashtra India

## Abstract

**Background:**

Griseofulvin, an antifungal drug, has recently been shown to inhibit proliferation of various types of cancer cells and to inhibit tumor growth in athymic mice. Due to its low toxicity, griseofulvin has drawn considerable attention for its potential use in cancer chemotherapy. This work aims to understand how griseofulvin suppresses microtubule dynamics in living cells and sought to elucidate the antimitotic and antiproliferative action of the drug.

**Methods:**

The effects of griseofulvin on the dynamics of individual microtubules in live MCF-7 cells were measured by confocal microscopy. Immunofluorescence microscopy, western blotting and flow cytometry were used to analyze the effects of griseofulvin on spindle microtubule organization, cell cycle progression and apoptosis. Further, interactions of purified tubulin with griseofulvin were studied *in vitro *by spectrophotometry and spectrofluorimetry. Docking analysis was performed using autodock4 and LigandFit module of Discovery Studio 2.1.

**Results:**

Griseofulvin strongly suppressed the dynamic instability of individual microtubules in live MCF-7 cells by reducing the rate and extent of the growing and shortening phases. At or near half-maximal proliferation inhibitory concentration, griseofulvin dampened the dynamicity of microtubules in MCF-7 cells without significantly disrupting the microtubule network. Griseofulvin-induced mitotic arrest was associated with several mitotic abnormalities like misaligned chromosomes, multipolar spindles, misegregated chromosomes resulting in cells containing fragmented nuclei. These fragmented nuclei were found to contain increased concentration of p53. Using both computational and experimental approaches, we provided evidence suggesting that griseofulvin binds to tubulin in two different sites; one site overlaps with the paclitaxel binding site while the second site is located at the αβ intra-dimer interface. In combination studies, griseofulvin and vinblastine were found to exert synergistic effects against MCF-7 cell proliferation.

**Conclusions:**

The study provided evidence suggesting that griseofulvin shares its binding site in tubulin with paclitaxel and kinetically suppresses microtubule dynamics in a similar manner. The results revealed the antimitotic mechanism of action of griseofulvin and provided evidence suggesting that griseofulvin alone and/or in combination with vinblastine may have promising role in breast cancer chemotherapy.

## Background

Griseofulvin (GF), an orally active antifungal drug, has been attracting considerable interest as a potential anticancer agent owing to its low toxicity and efficiency in inhibiting the proliferation of different types of cancer cells [1-4]. GF in combination with nocodazole was shown to potently inhibit tumor growth in athymic mice [1]. It induces apoptosis in several cancer cell lines [5] and it has also been proposed that GF can selectively kill the cancer cells sparing the normal healthy cells [3].

GF is known to inhibit the growth of fungal, plant and mammalian cells mainly by inducing abnormal mitosis and blocking the cells at G2/M phase of cell cycle [1-3,6-8]. Different organisms exhibit different degrees of sensitivity to GF owing to its differential affinity to different tubulins [6,9]. The concentration required to inhibit the growth of fungal cells is much lower than that required to inhibit the mammalian cells due to its higher affinity for fungal tubulin as compared to the mammalian tubulin [6,10-12]. GF has been reported to interact with tubulin [2,12-16] as well as microtubule associated proteins (MAPs) [13,17].

Recently, GF has been shown to suppress the dynamic instability of MAPs-free microtubules *in vitro *[2]. The spindle microtubules of HeLa cells treated with moderate concentrations of GF appeared to have nearly normal organization [2,18], while higher GF concentration caused depolymerization of the microtubules [2,16]. Based on the strong suppressive effects of GF on the microtubule dynamics *in vitro*, it was proposed that GF inhibits mitosis in HeLa cells by suppressing microtubule dynamics [2].

Although several studies suggested that tubulin is the primary target of GF [2,12,14-16], the binding site of GF in tubulin is yet unknown. Based on the findings that GF quenches tryptophan fluorescence of the colchicine-tubulin complex [12] and that colchicine can depolymerize GF-induced polymers of tubulin in the presence of MAPs at 4°C [13], it was suggested that GF binds at a site distinct than the colchicine binding site in tubulin [12].

In this study, we have identified two potential binding sites for GF in mammalian tubulin and provided a mechanistic explanation of how GF stabilizes microtubule dynamics. Further, the data suggested that GF inhibited mitosis in MCF-7 cells by suppressing the dynamicity of microtubules and that a population of the mitotically blocked cells escaped mitosis with misegregated chromosomes and eventually underwent apoptotic cell death.

## Methods

### Materials

GF, paclitaxel, vinblastine, mouse monoclonal anti-α tubulin IgG, rabbit monoclonal anti-γ tubulin IgG, alkaline phosphatase conjugated anti-mouse IgG, FITC (fluorescein isothiocyanate) conjugated anti-mouse IgG, fetal bovine serum, bovine serum albumin and Hoechst 33258 were purchased from Sigma (St. Louis, MO, USA). Mouse monoclonal anti-BubR1 antibody was purchased from BD Pharmingen (San Diego, USA). Rabbit polyclonal anti-Mad2 IgG was purchased from Bethyl laboratories (Montgomery, USA). Mouse monoclonal anti-Hec 1 IgG was purchased from Abcam (Cambridge, MA, USA). Anti-Mouse IgG alexa 568 conjugate and lipofectamine-2000 were purchased from Invitrogen (Carlsbad, CA, USA). Mouse monoclonal anti-p53 IgG, mouse monoclonal anti-p21 IgG, rabbit polyclonal anti-phosphohistone IgG and Annexin V apoptosis detection kit were purchased from Santa Cruz Biotechnology (CA, USA). All other reagents were of analytical grade.

### Effects of GF on MCF-7 cells and on the cell cycle progression

MCF-7 cells (1 × 10^5 ^cells/mL) were grown in 96-well tissue culture plates at 37°C for 24 h [19]. Then the medium was replaced with fresh medium containing vehicle (0.1% DMSO) or different concentrations of GF and the cells were grown for additional 48 h. Both attached and floating cells were harvested with the help of trypsin-EDTA solution and counted after staining with trypan blue [2]. MCF-7 cells were grown on glass coverslips in the absence or presence of different concentrations of GF for 24 h and the mitotic index was calculated by staining the chromosomes with Hoechst dye [19,20].

For determining the effect of GF on cell cycle progression, MCF-7 cells were grown for 48 h (one cell cycle) without or with different concentrations of GF. Samples were prepared as described recently [21]. DNA content of the cells was quantified in a flow cytometer (FACS Aria special order system, Becton Dickinson) and the cell cycle distribution was analyzed using the Modfit LT program [21].

### Immunofluorescence Microscopy

MCF-7 cells (5 × 10^4 ^cells/mL) were grown on glass coverslips in 24 well tissue culture plates for 24 h [19,20]. Then, the medium was replaced with fresh medium containing vehicle (0.1% DMSO) or different concentrations of GF (15, 30, 60 and 90 μM) and the incubation continued for further 24 or 48 h. The cells were then fixed with 3.7% formaldehyde at 37°C for 30 min and processed to visualize α tubulin, γ tubulin, p53, p21, BubR1, Mad2, Hec1 and phosphohistone [19,20].

### Analysis of the polymeric mass of tubulin

MCF-7 cells were incubated without or with different concentrations of GF (15, 30, 60 and 90 μM) for 48 h. The effect of GF on the amount of polymerized tubulin in MCF-7 cells was estimated by western blot analysis using anti α-tubulin antibody as described earlier [19] and the band intensities were estimated using Image J software. The band intensities for polymeric and soluble tubulin in the presence of different concentrations of GF were normalized with that of the vehicle-treated MCF-7 cells. The normalized band intensities were plotted against GF concentrations.

### Transfection of EGFP-α tubulin and measurement of microtubule dynamics

The effects of GF on the dynamic instability of individual microtubules in live MCF-7 cells were determined using EGFP-α tubulin as described recently [19,21]. Briefly, MCF-7 cells stably-expressing EGFP-α tubulin were incubated with vehicle or different concentrations of GF (5 and 15 μM) for 24 h. Individual microtubules on the peripheral region of the MCF-7 cells were observed using a 60 × water immersion objective in a FV 500 laser scanning confocal microscope (Olympus, Tokyo, Japan) [19,21]. The images were digitally magnified by 6 times and were acquired at 2 or 4 s intervals for a total period of 120-180 s using the FluoView software (Olympus, Tokyo, Japan). The lengths of the microtubules at different time points were calculated using Image-Pro Plus software (Media Cybernetics, Silver Spring, MD). The dynamic instability parameters were calculated as described earlier [2,19]. Transfection of MCF-7 cells with p53-GFP plasmid was done using lipofectamine-2000. Stably expressing p53-GFP MCF-7 cells were treated with different concentrations of GF (15-90 μM) and the localization of p53-GFP was studied using Nikon Eclipse TE-2000U fluorescent microscope. The images were analyzed with Image-Pro Plus software.

### Annexin V/propidium iodide staining

MCF-7 cells were grown in the absence and presence of different concentrations of GF (15, 30, and 60 μM) for 48 h. Control and treated cells were stained using the Annexin V/propidium iodide apoptosis kit, as described earlier [19] and the cells were examined under a fluorescence microscope.

### Effect of GF on BODIPY FL-vinblastine binding to tubulin

Goat brain tubulin (2 μM) was incubated without and with 100 μM GF, and with 50 μM vinblastine at 37°C for 45 min. Then, 2 μM fluorescent-tagged vinblastine (BODIPY FL-vinblastine) was added to each of the reaction mixtures and incubated for an additional 20 min in dark at 25°C. Fluorescence spectra of BODIPY FL-vinblastine were monitored by exciting at 490 nm.

### Effect of paclitaxel on GF binding to tubulin

Tubulin (10 μM) was polymerized in presence of 1 mM GTP, 5 mM MgCl_2_, and 10% DMSO without and with 15 μM paclitaxel at 37°C for 10 min. GF (50 and 100 μM) was added to the polymerization mixtures and then tubulin was allowed to polymerize for an additional 20 min. The polymers were pelleted down and washed with warm pipes buffer. The polymers were depolymerized on ice for 1 h using 0.01% NaOH in 25 mM pipes buffer. Fluorescence of GF was monitored at 437 nm by excitation at 330 nm. The concentration of GF in the pellet was then calculated using the fluorescence values of known concentrations. The concentration of protein in the pellet was measured using Bradford method [22].

### Docking of GF to tubulin

GF structure was obtained from the drugbank [23]. Protein coordinates were obtained from the protein data bank [24]. Modeler9v4 [25] was used for 3D structure modeling. The LigandFit [26] module of Discovery Studio 2.1 (Accelrys Inc., California) and Autodock4 [27] were used for docking. Naccess V2.1.1 was used for calculating solvent accessible surface area [28]. PyMol was used for rendering 3D structures [29].

The crystal structure of tubulin complexed with epothilone A [30] (pdb id 1TVK) was used for docking since this has the highest resolution (2.89 Å) among all the tubulin structures available to date. However, in this structure, residues 35A-60A (the suffix denotes the subunit) are disordered. This region was modeled using the corresponding region of β tubulin as the template. The (Φ,Ψ) values of all the residues in the modeled region are within the allowed region of the Ramachandran map [31]. In addition, the WHAT IF server [32] was used to check the modeled structure (Additional file 1, Table S1). Docking was performed for epothilone A as a control, using both the softwares. The root mean square deviation (RMSD) between the "predicted" and experimentally determined binding modes of epothilone A is 2 Å.

### Docking using Autodock4

Blind docking was performed by treating only GF as flexible. The entire protein was enclosed in a box with a grid spacing of 0.375 Å. Fifty docking jobs, each of hundred runs, were carried out using the Lamarckian genetic algorithm. Default values were used for all the parameters; g_eval was set to 2,500,000 (M). The resulting 5000 binding modes were clustered using an all-atom RMSD cutoff of 2 Å. Binding modes are ranked on the basis of cluster size. Highly populated (viz., size ≥ 50) clusters were chosen for further analysis. In these clusters, decrease in solvent accessible surface area of the protein on GF binding was calculated.

### Docking using LigandFit

Binding sites were defined on tubulin surface and GF was docked in each of these cavities. The ligand poses at these sites were selected on the basis of shape matching, using a cutoff distance of 3 Å between the ligand and the binding site residues. Binding modes are ranked on the basis of consensus scoring.

### Determination of combination Index (CI)

MCF-7 cells were treated with GF (10 and 15 μM), vinblastine (0.5 and 1 nM) or GF along with vinblastine for 48 h. The CI was calculated by Chou and Talalay method [33-35] using the equation:(1)

Where, *(D)1 *and *(D)2 *are the concentrations of drug 1 (GF) and drug 2 (vinblastine) in combination that produces a given effect, *(Dx)1 *and *(Dx)2 *are the concentrations of drug 1 and drug 2 that also produces the same effect when used alone.

The concentration of the drug that produces a particular effect *(Dx) *and the median dose (*Dm) *were calculated as described earlier [33-35]. CI <1 indicates synergism, CI = 1 indicates additivity, and CI >1 indicates antagonism.

## Results

### GF inhibited the proliferation of the MCF-7 cells and caused mitotic arrest

**Figure 1:**
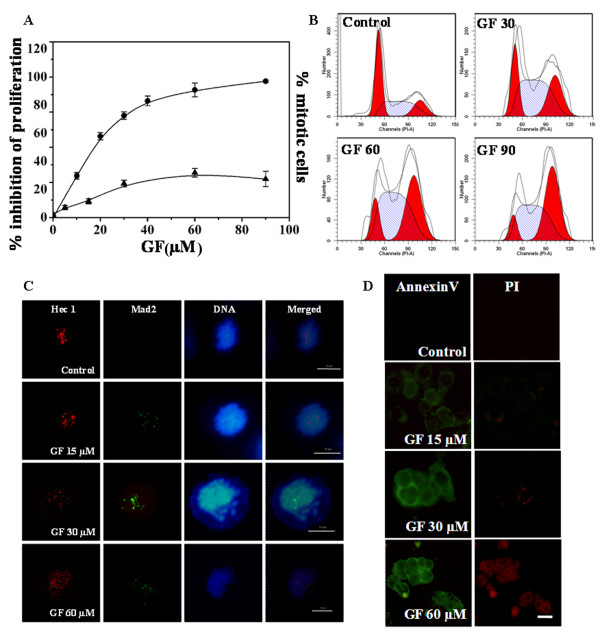
**GF inhibited MCF-7 cell proliferation, arrested cell cycle progression at G2/M phase, increased the accumulation of Mad2 at the kinetochores and induced apoptosis**. **(A) **The effect of GF on cell proliferation (closed circle) was determined by counting the cells after 48 h of incubation. The mitotic index (closed triangle) was calculated by Hoechst staining method after incubating the cells without or with GF for 24 h. Each experiment was performed four times. Data represent mean ± SD. **(B) **Flow cytometric analysis showing the effects of GF on the MCF-7 cell cycle **(C) **Effect of GF on Mad2 (green) localization. Staining was also done for a kinetochore protein Hec1 (red) **(D) **GF induced apoptosis in MCF-7 cells. Bars equal to 10 μm

GF inhibited the proliferation of MCF-7 cells with an IC_50 _of 17 ± 2 μM after 48 h of incubation (one cell cycle) (Figure 1A). The proliferation was inhibited by 73 and 91% in the presence of 30 and 60 μM of GF, respectively. GF arrested MCF-7 cells at mitosis in a concentration dependent manner (Figure 1A). For example, 2, 11, and 32% of the cells were found in different stages of mitosis in the absence or presence of 15 and 60 μM of GF, respectively.

Flow cytometry analysis suggested that GF inhibited cell cycle progression of MCF-7 cells in the G2/M phase. For example, 16, 26, 36 and 46% of cells were found to be in the G2/M phase in the absence or presence of 30, 60 and 90 μM of GF, respectively (Figure 1B). Further, GF-treatment increased the number of phosphohistone H3 positive MCF-7 cells in a concentration dependent manner (Additional file 1, Figure S1). For example, 2.9 ± 0.2%, 12.3 ± 1.3% and 28.2 ± 3.3% of the cells were found to be phosphohistone H3 positive in the absence and the presence of 15 and 60 μM GF, respectively suggesting that GF treatment increased the number of mitotic cells.

### GF perturbs microtubule-kinetochore attachment and the tension across the kinetochores in MCF-7 cells

Spindle assembly check point proteins like BubR1 and Mad2 are known to sense the tension across the kinetochores and microtubule attachment to the kinetochores [36,37]. The position of the kinetochores has been shown by Hec1 protein (Figure 1C). In the presence of GF, Mad2 was found to localize at the kinetochores (Figure 1C). BubR1 was also found to be localized to the kinetochores in GF treated cells (Additional file 1, Figure S2). The results indicated that GF disrupts the attachment of microtubules to kinetochores and also disturbs the tension across the kinetochores.

### GF induced apoptotic cell death in MCF 7 cells

Although control MCF-7 cells remained viable after 48 h, GF-treated cells were found at various stages of apoptosis. GF (15 and 30 μM) treated cells stained positive for Annexin V and weakly stained with propidium iodide indicating that the cells were in early or mid apoptotic phase. Cells treated with 60 μM GF stained positive for both Annexin V and propidium iodide indicating them to be in the late apoptotic phase (Figure 1D).

### GF induced the nuclear localization of p53 and p21

**Figure 2:**
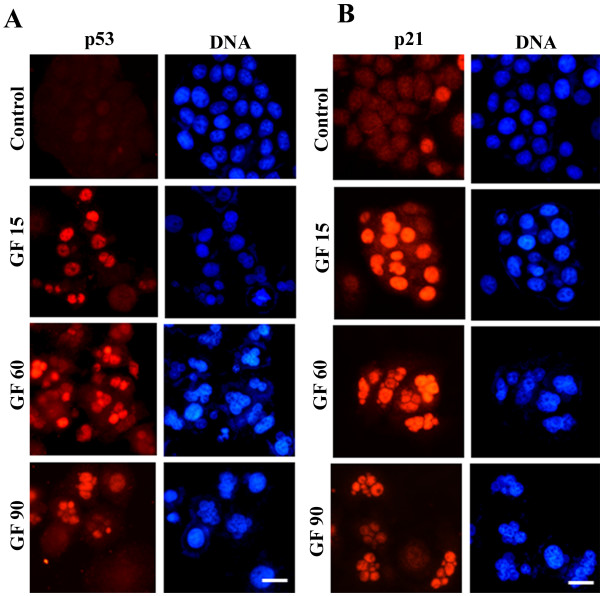
**GF treatment increased the nuclear accumulation of p53 and p21**. MCF-7 cells were incubated with different concentrations of GF (15-90 μM) for 48 hours, fixed and processed to visualize either (A) p53 (red) and DNA (blue) or (B) p21 (red) and DNA (blue). Bar equals to 20 μm.

GF treatment strongly increased the nuclear localization of p53 in MCF-7 cells (Additional file 1, Table S2 and S3). For example, 3, 39, 30 and 35% of the MCF-7 cells were found to have p53 translocated into the nucleus after 48 h incubation with vehicle, 15, 30, and 60 μM of GF, respectively (Figure 2A, Additional file 1, Table S3). Consistent with the previous studies [2,3], GF was found to induce multipolarity in MCF-7 cells. GF treatment also induced the formation of fragmented nuclei/multiple nuclei in MCF-7 cells. For example, 31 ± 7 and 50 ± 12% of the interphase cells were with fragmented nuclei after 24 and 48 h of incubation with 90 μM GF, respectively while, only 2 ± 1.2 and 1 ± 0.7% of vehicle-treated cells had fragmented nuclei (Additional file 1, Table S2 and S3).

Interestingly, the cells containing fragmented nuclei exhibited increased accumulation of p53 in the nucleus as compared to the mononucleated interphase cells of the same treatment group (Figure 2A, Additional file 1, Table S2 and S3). For example, 11 ± 1% of mononucleated cells and 63 ± 3% of the interphase cells with fragmented nuclei were found to have nuclear translocation of p53 after 24 h incubation with 15 μM GF. The number of mononucleated cells containing p53 decreased with increasing drug concentration. However, the number of cells containing fragmented nuclei increased with increasing drug concentration and more than 65% of these cells containing fragmented nuclei were found to contain enhanced concentration of p53 in their nuclei. Similarly, MCF-7 cells transfected with GFP-p53 also showed enhanced nuclear localization of GFP-p53 and fragmentation of nuclei upon GF treatment (Additional file 1, Figure S3; Additional file 1, Table S4). For example, in the presence of 15 μM GF, 42% of the cells with fragmented nuclei and 12% of the mononucleated cells accumulated GFP-p53 in their nuclei while only 2% of the mononucleated control cells (vehicle-treated) had p53 localized in the nuclei. p21, the downstream target of p53, was also observed to exhibit similar localization pattern as p53 (Figure 2B). In the presence of 60 and 90 μM GF, most of the cells contained multiple nuclei and enhanced concentration of p21 in their nuclei (Figure 2B).

### GF affected microtubule and chromosome organization in MCF-7 cells

**Figure 3:**
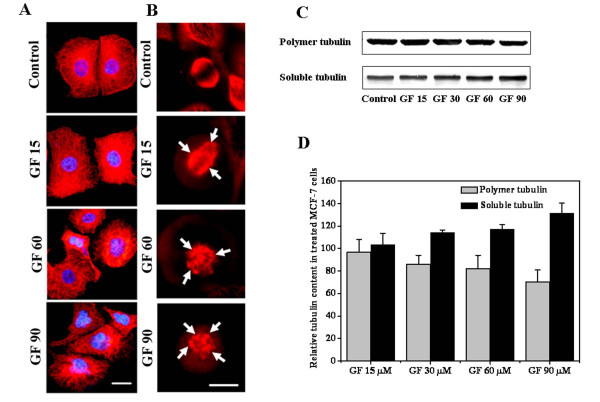
**Effects of GF on the microtubules of MCF-7 cells**. MCF-7 cells were incubated without or with different concentrations of GF for 48 h. (A) Microtubules (red) and chromosomes (blue) of interphase cells or (B) Microtubules (red) of metaphase cells are shown. Arrows point towards the poles. Bar equals to 20 and 10 μm for an interphase and a metaphase cell, respectively. (C) Effects of GF on the polymer mass of tubulin in MCF-7 cells. (D) Band intensities of the polymeric and soluble tubulin fractions in MCF-7 cells treated with different GF concentrations relative to the vehicle-treated cells are shown. The experiment was performed three times. Data represent mean ± SD.

GF (15 μM) did not alter the interphase microtubular network as they were found to be similar to the vehicle (0.1% DMSO) treated cells. A moderate depolymerization of interphase microtubules was observed in the presence of 30 μM GF (data not shown). Higher concentrations (60 and 90 μM) of GF caused depolymerization of the interphase microtubules and also increased the number of cells containing fragmented nuclei (Figure 3A). The effects of GF on the spindle microtubules were more clearly noticeable than its effects on the interphase microtubules (Figure 3B). In the presence of 15 μM GF, few chromosomes were located near the spindle poles and few chromosomes were lagging behind and were not positioned at the metaphase plate. GF (≥ 30 μM) caused a significant depolymerization of the spindle microtubules. Spindles were found to be multipolar in the presence of high concentrations (≥ 30 μM) of GF (Figure 3B). The chromosomes looked like rounded mass without proper alignment at the metaphase plate (Additional file 1, Figures S1 and S4).

GF treatment caused the formation of multiple poles in MCF-7 cells as observed by γ tubulin staining. In the presence of 15 μM GF, the distance between the two poles was significantly decreased; however, cells containing multiple centrosomes were found to increase with increasing concentrations of GF (Additional file 1, Figure S4).

Western blot analysis showed that GF (15 μM) did not significantly change the polymeric mass of tubulin. GF decreased the polymeric tubulin mass by 14%, 18% and 30% in the cells treated with 30, 60 and 90 μM of GF, respectively as compared to the vehicle-treated cells (Figure 3C and 3D). Similarly, the soluble tubulin mass was found to be increased by 13%, 17% and 31% in the cells treated with 30, 60 and 90 μM GF, respectively as compared to the vehicle-treated cells (Figure 3C and 3D).

### GF suppressed the dynamic instability of microtubules in live MCF-7 cells

**Figure 4:**
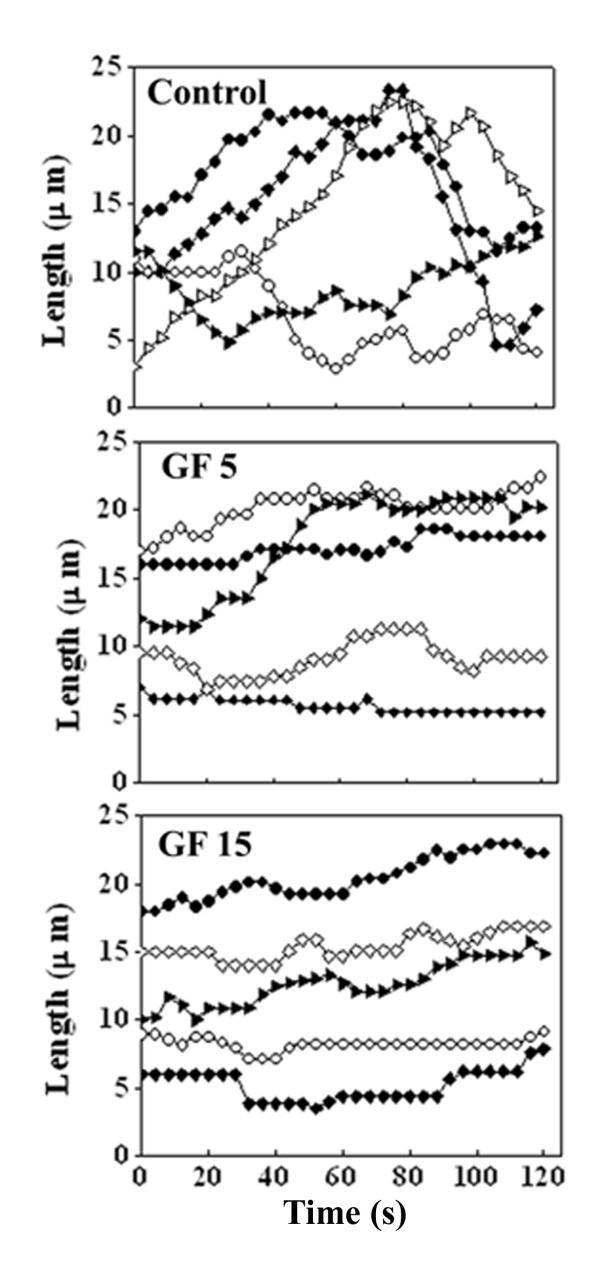
**Life history traces of individual microtubules in the absence or presence of 5 and 15 μM of GF are shown**.

Microtubules in the vehicle-treated MCF-7 cells were highly dynamic and GF (15 μM, ~IC_50_) strongly suppressed the dynamic instability of individual microtubules in MCF-7 cells (Figure 4). GF (15 μM) reduced the growing and shortening rates of microtubules in MCF-7 cells by 46 and 48.5% respectively (Table 1). The mean growth length and shortening length of the microtubules were reduced by 50 and 58%, respectively. GF (15 μM) reduced the time microtubules spent in growing and shortening phases by 53 and 31%, respectively while increased the time microtubule spent in the pause state (neither growing nor shortening detectably) by 103% compared to the control microtubules.

**Table 1:** Effects of GF on the dynamic instability parameters of individual MTs

	GF (μM)		
	
	0	5	15
**Rate (μm/min)**			
Growing	18.5 ± 4	12.0 ± 2.2 ^a^	10.0 ± 2.0^a^
Shortening	19.6 ± 5	12.7 ± 4.6 ^a^	10.1 ± 2.6^a^
**Length Change (μm)**			
Growth length	2.0 ± 0.8	1.4 ± 0.6^b^	1.0 ± 0.4^a^
Shortening length	2.4 ± 0.7	1.4 ± 1^c^	1.0 ± 0.3^a^
**% Time in phase**			
Growing	44.0 ± 10	33.0 ± 10^a^	20.5 ± 6^a^
Shortening	25.5 ± 10	14.5 ± 5^a^	17.5 ± 4^b^
Pause	30.5 ± 9	52.5 ± 9^a^	62.0 ± 9^a^
**Frequency (events/min)**			
Catastrophe	4.0 ± 2	1.6 ± 0.5^a^	2.3 ± 0.8^b^
Rescue	10.0 ± 3	10.0 ± 4^d^	10.0 ± 2.6^c^
**Frequency (events/μm)**			
Catastrophe	0.40 ± 0.2	0.5 ± 0.3^d^	1.1 ± 0.5^a^
Rescue	0.6 ± 0.3	0.9 ± 0.5^b^	1.1 ± 0.3^a^
**Dynamicity (μm/min)**	13.7 ± 4	5.7 ± 2^a^	3.8 ± 1.2^a^

Data are average ± SD, n = 22 MTs in control and n = 21 MTs in 5 & 15 mM GF treated cells; a, P < 0.001; b, P < 0.01; c, P < 0.05; d, statistically not significant

The transition of a microtubule end from a shortening phase to a growth or a pause phase is called a rescue while the transition of a microtubule from a growing or a pause to a shortening phase is called a catastrophe [38]. GF (15 μM) increased the length based rescue and the catastrophe frequencies (events/μm) by 83 and 175%, respectively compared to the control microtubules. The time based rescue frequency (events/min) was not significantly different from the control treatment while the time based catastrophe frequency (events/min) was found to be decreased by 42.5% in the 15 μM GF treated samples compared to the control cells. GF (15 μM, ~IC_50_) reduced the dynamicity (dimer exchange per unit time) of the microtubules by 72% compared to the control microtubules (Table 1). In addition, 5 μM of GF also considerably suppressed the dynamic instability of individual microtubules by reducing the rates of growing and shortening, and increasing the time microtubule spends in the pause state. For example, it inhibited the dynamicity of microtubules by 58%.

### Docking analysis and in vitro competition experiments indicated that GF shares binding site in tubulin with paclitaxel

**Figure 5:**
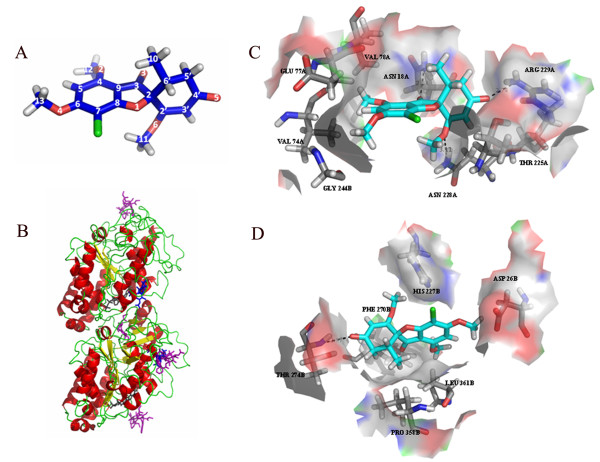
**Mode of interaction of GF with tubulin**. (A) Structure of GF. The conformation of the O-Me groups are chosen arbitrarily. Color code: carbon, blue; oxygen, red; hydrogen, grey; chlorine, green. (B) Cartoon rendering of the αβ tubulin heterodimer (PDB id 1TVK) showing the location of the binding sites for colchicine, paclitaxel and vinblastine (purple), GTP and GDP (black) and GF (blue). The "upper" domain is α tubulin and the "lower" domain is β tubulin. In this view, GTP and GDP are partly hidden. Vinblastine binds at the inter-dimer (αβ)-interface and hence, is shown both at the top and bottom. Colchicine binds at the intra-dimer interface (αβ) and paclitaxel binds to the β-subunit. The binding sites for GF, one at the interface (site A) and the other overlapping with that of paclitaxel (site B), are predicted by docking; all others are based on X-ray crystallographic studies. (C) and (D) Representative binding modes of GF in site A (C) and site B (D) obtained by docking. Atoms are colored by element type except for carbon (cyan for GF and grey for binding site residues). Hydrophobic residues constituting the binding pocket and polar residues within 3 Å of GF are shown.

Autodock4 and LigandFit were used to identify the binding site(s) of GF (Figure 5A) in tubulin and only the binding sites that were identified by the two methods were considered as putative binding sites. Two binding sites were identified for GF (Figure 5B). Site A is at the intra-dimer (αβ) interface and distinctly away from GDP, colchicine and vinblastine binding sites (Additional file 1, Table S5). This site is approximately equidistant from both GTP and colchicine binding sites. Site B overlaps with paclitaxel binding site.

GF was also docked using LigandFit with Nsave 20 to determine the binding interactions at these two sites. The binding poses were clustered using RMSD cutoff of 1 Å. Both the predicted sites are primarily hydrophobic. This can be expected from the chemical nature of GF, which has only hydrogen bond acceptors and no hydrogen bond donor (Figure 5A). Further, the three oxygen atoms from the O-Me groups and the ring oxygen atom can accept hydrogen bonds in only certain restricted directions due to steric reasons. The residues Val74A, Glu77A, Val78A, Thr225A, and Gly244B (suffixes A and B are subunit identifiers) are involved in hydrophobic interaction at Site A and the residues Asp26B, His227B, Phe270B, Pro358B, and Leu361B are involved at Site B (Figure 5C, D).

GF was found to be incorporated into the microtubules. For example, 0.15 ± 0.02 and 0.24 ± 0.04 moles of GF per tubulin dimer were found to be incorporated in microtubules in the presence of 50 μM and 100 μM of GF, respectively. Paclitaxel (15 μM) reduced the incorporation of GF in microtubules to 0.1 ± 0.02 and 0.15 ± 0.02 moles of GF per tubulin dimer. The decrease in GF incorporation in microtubules in the presence of paclitaxel was significantly different at 0.001 level. Since the binding affinity of GF with tubulin is very weak with a dissociation constant of 300 ± 12 μM [2], the incorporation ratios of GF are expected to be low at these concentrations. The results indicated that the binding site of GF in tubulin may overlap with the paclitaxel site.

The preincubation of tubulin with GF did not affect the binding of fluorescent-tagged vinblastine to tubulin indicating that GF binds at a site different from that of the vinblastine site (Additional file 1, Figure S5). Moreover, GF did not affect the binding of TNP-GTP (a fluorescent analogue of GTP) to tubulin and also quenched the intrinsic tryptophan fluorescence of GTP-bound tubulin (data not shown). The results support the finding of the docking analysis that GF binds to tubulin at a site, which is distinct from the GTP binding site.

### GF in combination with vinblastine synergistically inhibited the proliferation of MCF-7 cells

**Figure 6:**
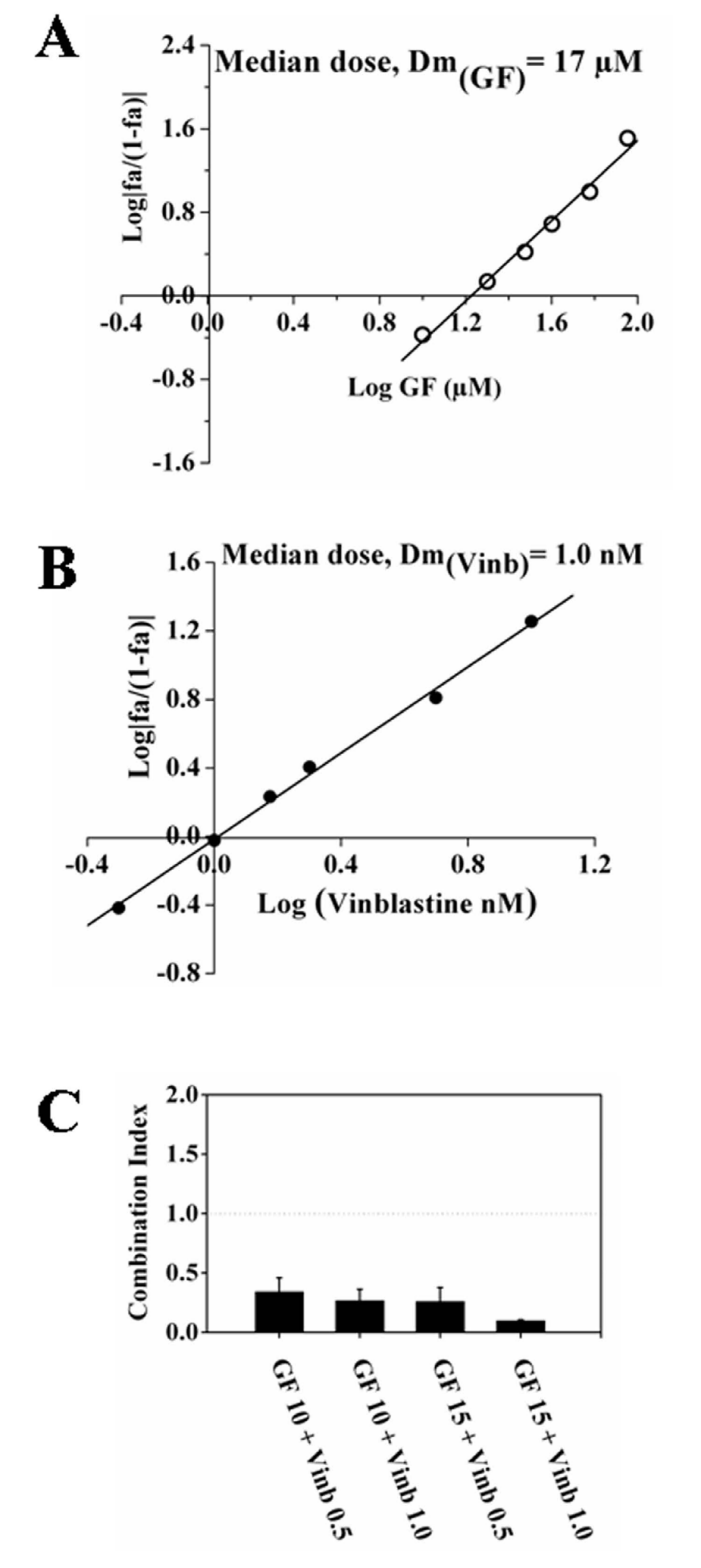
**Combination of GF and vinblastine exerted synergistic effects in inhibiting MCF-7 cell proliferation**. The median effect plots for the inhibition of cell proliferation in the presence of GF (A) and vinblastine (B) are shown. (C) The effects of the combination of GF and vinblastine (Vinb) on MCF-7 cell proliferation after 48 h treatment are shown as combination index. Data are average of four experiments and error bars represent SD.

GF and vinblastine inhibited the proliferation of MCF-7 cells with a median dose of 17 μM and 1 nM, respectively (Figure 6A and 6B). The combination of 10 μM GF with 0.5 and 1 nM vinblastine inhibited the proliferation of MCF-7 cells by 84 and 92%, respectively. Using combinatorial analysis [33], CI values for the combination of 10 μM GF with 0.5 or 1 nM vinblastine were estimated to be 0.34 ± 0.1 and 0.27 ± 0.1, respectively while the combination of 15 μM GF with 0.5 and 1 nM vinblastine yielded CI values of 0.26 ± 0.1 and 0.1 ± 0.01, respectively (Figure 6C). The results suggested that GF and vinblastine exerted synergistic effects on MCF-7 cell proliferation.

Cells treated with either GF 10 μM or vinblastine 0.5 nM exhibited nearly normal bipolar mitotic spindles (Additional file 1, Figure S6). When used alone, neither GF (15 μM) nor vinblastine (1 nM) disrupted the organization of the mitotic spindle microtubules in most of the cells; however, a small population of cells had disorganized spindles. The combined treatment of GF and vinblastine strongly disrupted the organization of the spindle microtubules (Additional file 1, Figure S6).

MCF-7 cells treated with GF (10 and 15 μM), or vinblastine (0.5 and 1 nM) exhibited nearly normal interphase microtubule network as observed in the control cells. The combination of 10 μM GF with either 0.5 or 1 nM vinblastine caused significant depolymerization of the interphase microtubules. The combination of 15 μM GF with either 0.5 or 1 nM vinblastine strongly depolymerized interphase microtubules (Additional file 1, Figure S7). The finding that the combination of GF and vinblastine induced stronger depolymerizing effects on the spindles and interphase microtubules than either of the drugs used alone provided a mechanistic basis for their synergistic activity on cell proliferation.

## Discussion

GF (≤ IC_50_) strongly suppressed the dynamics of individual microtubules in live MCF-7 cells without detectably altering the microtubule network. However, at higher concentrations, GF induced significant depolymerization of both the mitotic spindles and the interphase microtubules. The suppressive effects of GF on the dynamic instability of interphase microtubules of MCF-7 cells were found to be qualitatively similar to its effects on the bovine brain microtubules *in vitro *[2]. In our studies, the IC_50 _of GF for the inhibition of cell proliferation has been found to be 17 ± 2 μM, which is comparable to some of the anticancer agents that are undergoing clinical trials. For example, estramustine (clinical trials.gov identifier NCT00151086), curcumin (NCT00094445) and noscapine (NCT00912899) inhibit the proliferation of MCF-7 cells with the IC_50 _of 5 ± 1 μM [21], 12 ± 0.6 μM [39] and 39.6 ± 2.2 μM [40], respectively.

GF was also found to be incorporated into the microtubules in high stoichiometry (0.24 molecules of GF per tubulin dimer) suggesting that GF binds along the length of the microtubules. Like paclitaxel [41,42], GF did not strongly influence the time based catastrophe and rescue frequencies. GF suppressed the dynamics by reducing the rate and extent of the growing and shortening excursions and increasing the time microtubule spent in the pause state. The docking studies indicated that GF has two potential binding sites in tubulin; one of these sites is overlapping with the paclitaxel binding site and the other lies at the interface of αβ tubulin, which is distinct from the GDP, vinblastine, and colchicine sites. The docking analysis is consistent with the findings that GF neither binds to the colchicine site [12] nor the vinblastine site in tubulin (Additional file 1, Figure S5; Additional file 1, Table S5). A competition experiment with paclitaxel showed that paclitaxel reduced the binding of GF to tubulin in microtubules supporting the computational analysis data that GF binding site partially overlaps with the paclitaxel site in tubulin. Paclitaxel is known to stabilize microtubule dynamics [42] and like GF, paclitaxel has been shown to bind along the length of microtubules [41]. Therefore, it is logical to propose that GF stabilizes microtubules dynamics by binding to tubulin in the paclitaxel site. It is likely that at higher concentrations GF binds to tubulin in the putative second site, which is located at the intra-dimer interface and induces microtubule depolymerization.

Defects in the microtubule-kinetochore attachment and the tension across the sister kinetochores are sensed by the check point proteins and the accumulation of check point proteins at the kinetochore region is thought to prevent the cells to enter into the anaphase until the defects are corrected [20,21,36,37]. The enhanced localization of BubR1 and Mad2 on the kinetochores upon GF treatment suggested that GF inhibited the extinction of checkpoint proteins from the kinetochores (Figure 1C; Additional file 1, Figure S2). The accumulation of BubR1 and Mad2 at the kinetochores activated the mitotic checkpoint and arrested the cells at mitosis. These mitotically blocked cells either undergo apoptosis or make an aberrant mitotic exit without cytokinesis resulting in cells with fragmented nuclei, which eventually undergo apoptosis [3,43]. The presence of multiple poles in GF treated cells probably results in improper chromosome segregation leading to the formation of multiple nuclei. The FACS analysis did not show an increase in DNA content (> 4N) indicating that multiple nuclei in the cells are indeed due to the improper chromosome segregation and not due to the multiplication of DNA. GF treatment caused a strong increase in multipolar mitosis leading to the formation of fragmented nuclei of varying sizes in MCF-7 cells. Most (> 60%) of these cells with fragmented nuclei had much higher accumulation of p53 as compared to the mononucleated cells suggesting that the cells that committed aberrant exit from the mitotic block with fragmented nuclei underwent apoptosis. Therefore, GF may induce apoptosis in MCF-7 cells via a series of concerted events, which includes formation of multipolar spindles, fragmentation of the nucleus, nuclear accumulation of p53, and finally p53 dependent induction of apoptosis. Cells treated with higher GF concentrations (> 30 μM) had hyper amplified centrosomes (Figure 3B; Additional file 1, Figure S4) and completely disorganized multipolar mitosis (Figure 3B). As a result, GF induced a concentration and a time dependent increase in the number of cells containing fragmented nuclei. The organization of centrosomes plays an important role in the successful completion of mitosis. Microtubule interacting drugs like paclitaxel, nocodazole, vinblastine and podophyllotoxin were shown to affect the organization of the centrosomes and cause functional impairment [44]. It has been found that GF inhibited the centrosomal clustering without interfering with the functions of NuMA and dynein and it was indicated that the alteration of the interphase microtubule stability by GF might be the reason for the inhibition of centrosomal clustering [3]. The evidence presented in this study strongly suggested that the kinetic suppression of microtubule dynamics induces mitotic irregularities and nuclear accumulation of p53.

Drugs having adverse side effects can be successfully used for chemotherapy if their effective doses are reduced significantly. Moreover, the use of combination of two or more drugs reduces the chances of survival of the resistant cancer cells [44]. For example, the use of haloperidol in combination with vinblastine reversed the resistance of K562/VBL cells to vinblastine [45]. In this work, we have found that the combination of GF and vinblastine exhibited strong synergistic effects on the inhibition of proliferation of MCF-7 cells.

## Conclusions

The study provided mechanistic insights into the antiproliferative action of GF on MCF-7 cells. GF arrested MCF-7 cells at mitosis and perturbed microtubule dynamic instability in cells, thus driving the cells to undergo programmed cell death. It exerts these effects by binding along the length of the microtubules, possibly at the paclitaxel site. Its potential activity against breast cancer cells has been explored for the first time and also the combination studies with vinblastine show that the two drugs together may successfully be used in the treatment of breast cancer.

## Abbreviations

GF: Griseofulvin; IC_50_: half-maximal proliferation inhibitory concentration; MAPs: microtubule associated proteins; FITC: fluorescein isothiocyanate; EGFP: enhanced green fluorescent protein; RMSD: root mean square deviation; CI: combination index; TNP-GTP: 2',3'-O-(2,4,6-trinitrocyclohexadienylidene) guanosine 5'-triphosphate

## Competing interests

The authors declare that they have no competing interests.

## Authors' contributions

KR performed microtubule dynamics studies and the cell culture experiments, analyzed the data and contributed in writing the manuscript. BJ performed docking studies and in vitro experiments and contributed in writing the manuscript. JA performed the cell culture experiments and contributed in writing the manuscript. PS performed flow cytometry and contributed in manuscript preparation. PVB provided help for docking studies, manuscript preparation and scientific discussions. DP provided the resources for the work, helped in data analysis and wrote the manuscript. All the authors have read and approved the final version of the manuscript.

## Pre-publication history

The pre-publication history for this paper can be accessed here:

http://www.biomedcentral.com/1471-2407/10/213/prepub

## Supplementary Material

Additional file 1 **Supplemental material**. Additional Figures and tables griseofulvin 12-5-2010 Click here for file
