# Supplementary material for: Griseofulvin stabilizes microtubule dynamics, activates p53 and inhibits the proliferation of MCF-7 cells synergistically with vinblastine
Source: BMC Cancer. 2010 May 19;10:213. doi: 10.1186/1471-2407-10-213 (PMC2885362; doi:10.1186/1471-2407-10-213)
Supplement: Additional file 1 — Supplemental material. Additional Figures and tables griseofulvin 12-5-2010 [file 1471-2407-10-213-S1.PDF]

Figure S1.

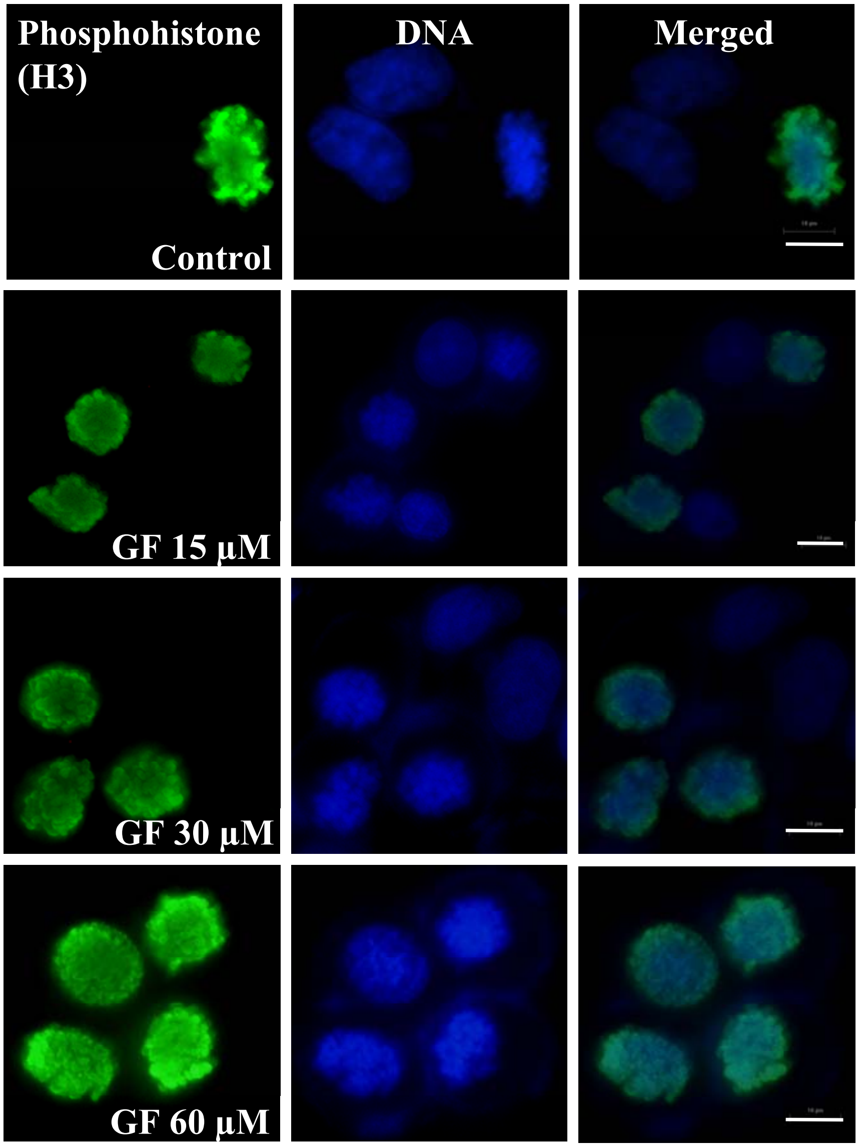

Figure S1. Griseofulvin caused M phase arrest in MCF-7 cells. MCF-7 cells were treated with the vehicle control (0.1 % DMSO) or with griseofulvin (15- 60 μM) for 48 h. The cells were then stained with anti-phoshphohistone (H3) antibody and Hoechst 33258 dye, and were observed under fluorescence microscope. Bars equal to 10 μm.

Figure S2.

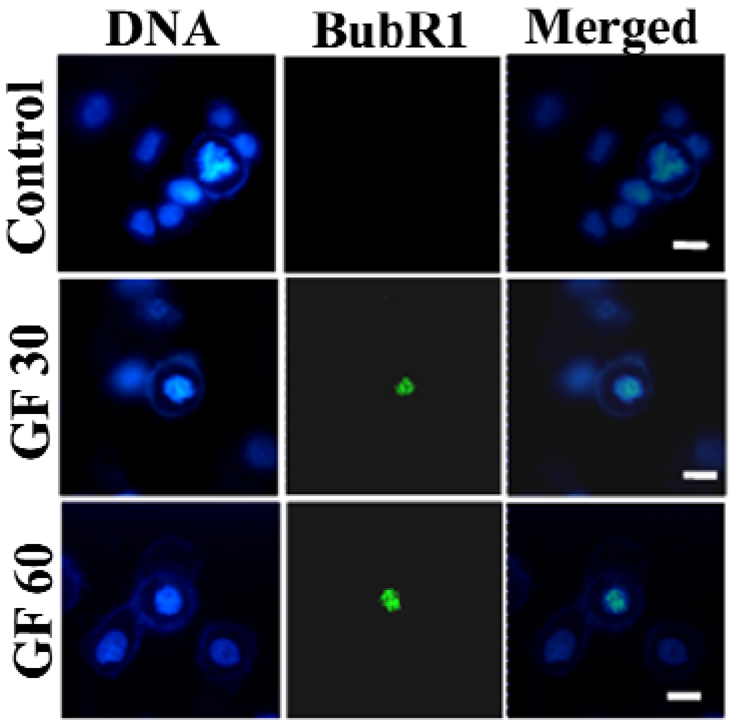

Figure S2. Griseofulvin caused an increase in the accumulation of BubR1 at the kinetochores of MCF-7 cells. MCF-7 cells were treated with the vehicle control (0.1 % DMSO) or with griseofulvin (30 and 60 μM) for 48 h. The cells were then stained with anti-BubR1 antibody and Hoechst 33258 dye, and were observed under fluorescence microscope. Bars equal to 10 μm.

Figure S3.

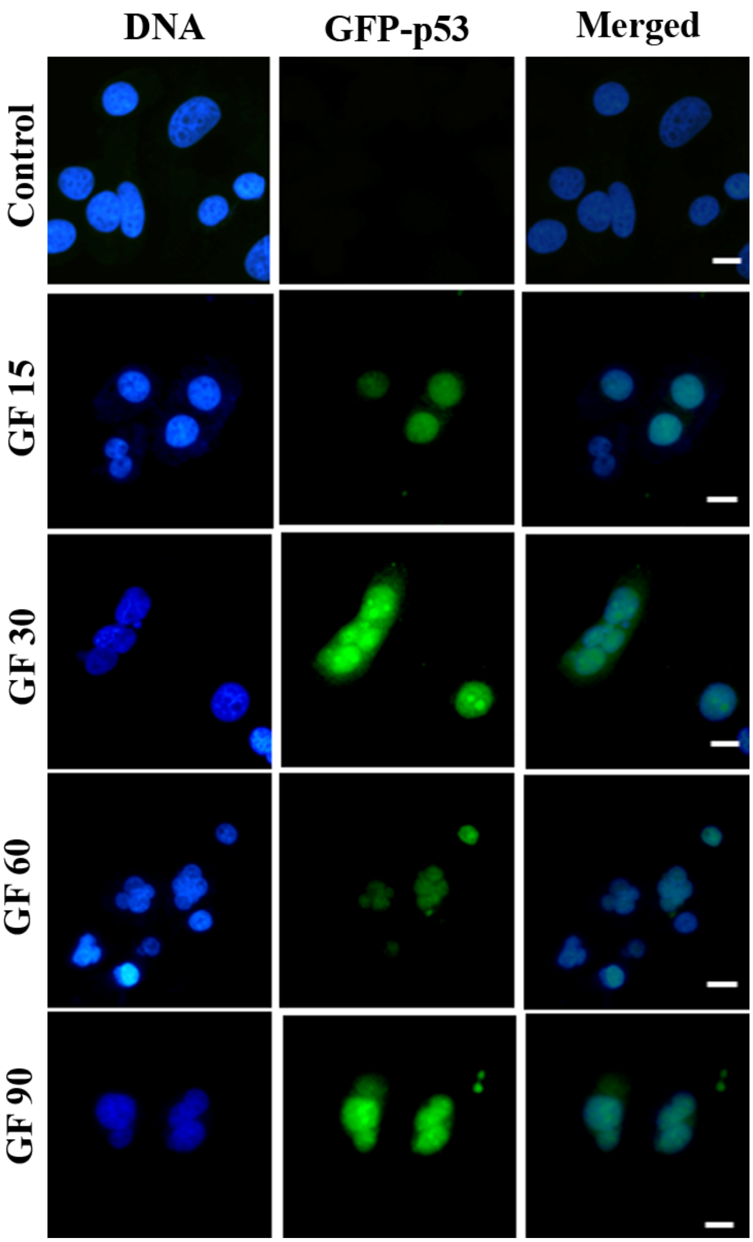

Figure S3. Griseofulvin caused nuclear accumulation of GFP-p53 in MCF-7 cells. MCF-7 cells were treated with the vehicle control (0.1 % DMSO) or with griseofulvin (15- 90 μM) for 48 h and were observed under fluorescence microscope. Bars equal to 10 μm.

Figure S4.

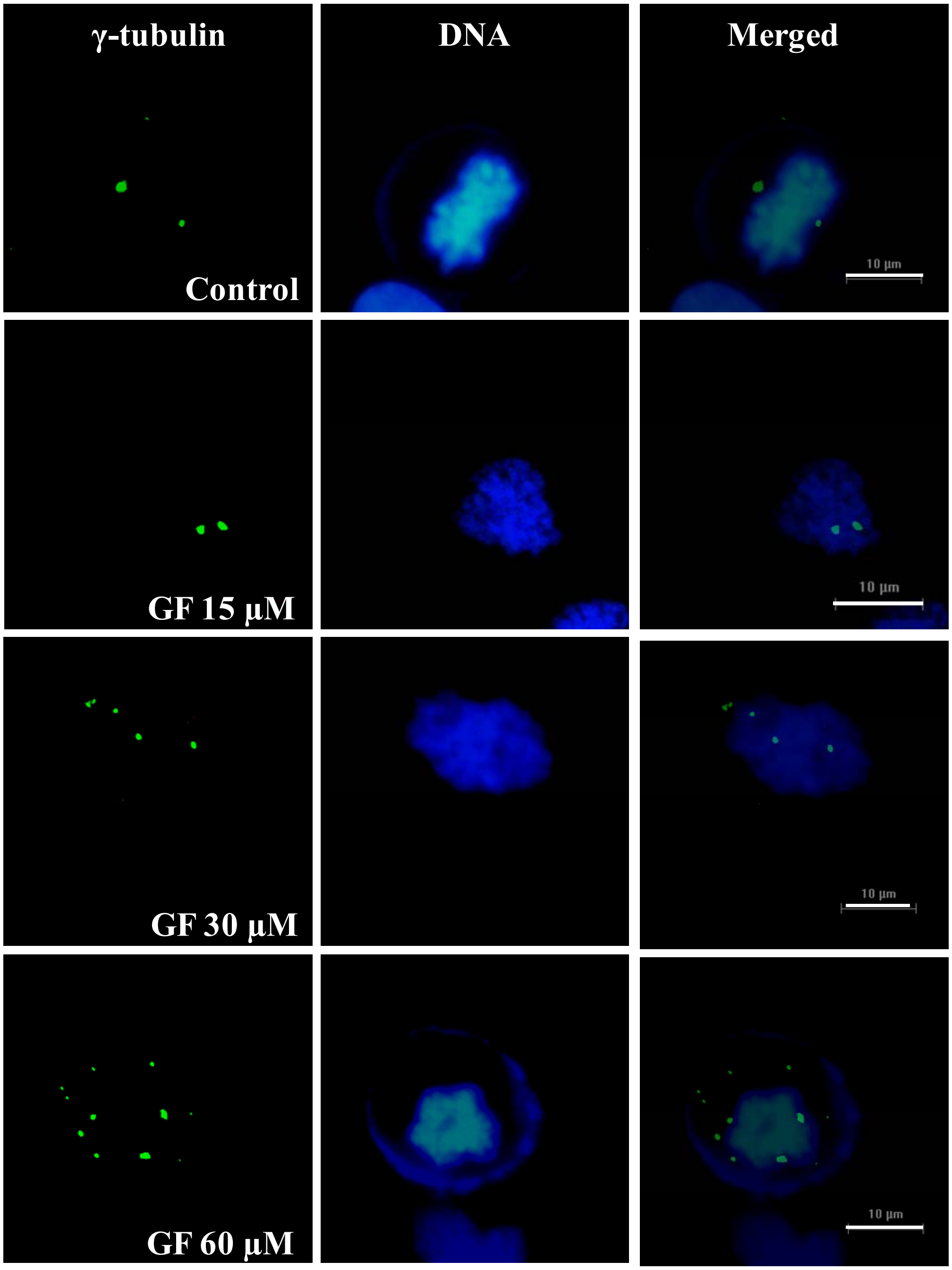

Figure S4. Griseofulvin induced the formation of multipolar spindles in MCF-7 cells. MCF-7 cells were treated with the vehicle control (0.1 % DMSO) or with griseofulvin (15- 60 µM) for 48 h. The cells were then stained with anti-gamma tubulin antibody and Hoechst 33258 dye, and were observed under fluorescence microscope. Bars equal to 10 µm.

Figure S5.

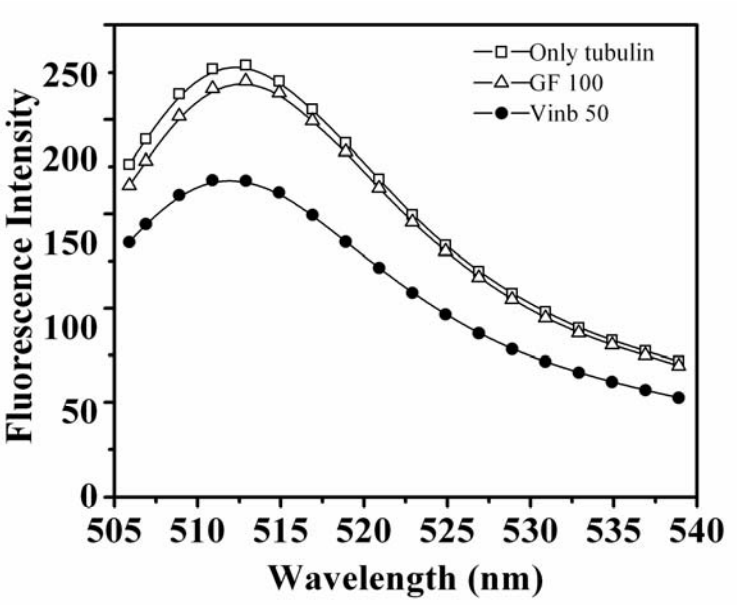

Figure S5. Griseofulvin did not affect the binding of BODIPY FL-vinblastine to tubulin. Fluorescence spectrum of BODIPY FL-vinblastine (50 μM) bound to tubulin in absence and presence (100 μM) of GF, and in presence of 50 μM vinblastine (Vinb).

Figure S6.

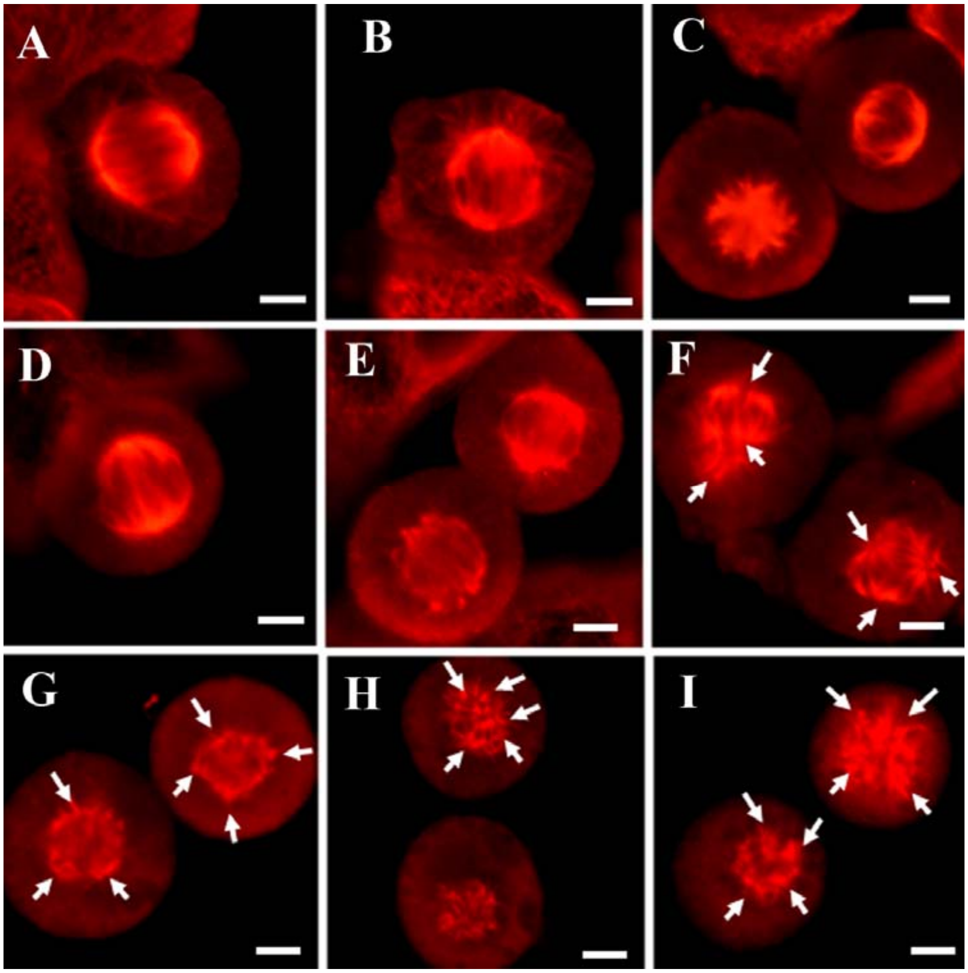

Figure S6. Effects of griseofulvin, vinblastine and the combination of griseofulvin and vinblastine on the spindle organization. MCF-7 cells were treated with the vehicle control or griseofulvin or vinblastine or griseofulvin and vinblastine added together and incubated for 48 h and then fixed and processed to visualize tubulin (red). The arrows point towards the poles. The spindle microtubules of the control (0.1 % DMSO) (A), griseofulvin 10 μM (B), griseofulvin 15 μM (C), vinblastine 0.5 nM (D), vinblastine 1.0 nM (E), griseofulvin 10 μM and vinblastine 0.5 nM (F), griseofulvin 10 μM and vinblastine 1 nM (G), griseofulvin 15 μM and vinblastine 0.5 nM (H) and griseofulvin 15 μM and vinblastine 1.0 nM (I) treated cells are shown. Bars equal to 5 μm.

Figure S7.

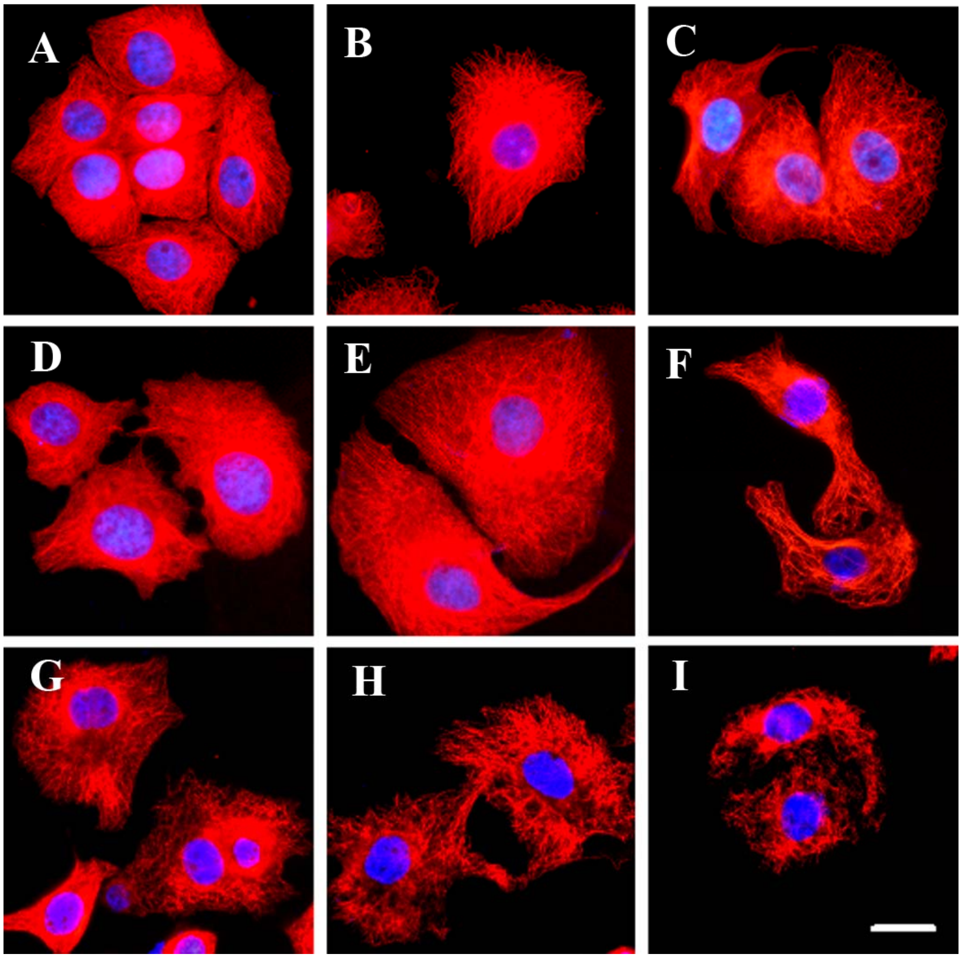

Figure S7. Effects of griseofulvin, vinblastine and the combination of griseofulvin and vinblastine on the interphase microtubule organization. MCF-7 cells were treated with the vehicle control or griseofulvin or vinblastine or griseofulvin and vinblastine added together and incubated for 48 h and then fixed and processed to visualize tubulin (red) and DNA (blue). The merged images of the organization of the interphase microtubules and DNA of the control (0.1 % DMSO) (A), griseofulvin 10 µM (B), griseofulvin 15 µM (C), vinblastine 0.5 nM (D), vinblastine 1.0 nM (E), griseofulvin 10 µM and vinblastine 0.5 nM (F), griseofulvin 10 µM and vinblastine 1 nM (G), griseofulvin 15 µM and vinblastine 0.5 nM (H) and griseofulvin 15 µM and vinblastine 1.0 nM (I) treated cells are shown. Bar equals 20 µm.

Table S1. RMS Z-scores reported by WHAT IF web server

	Before modeling	Modeled structure
Bond lengths	0.436	0.906
Bond angles	0.724	0.887
Omega angle restraints	0.039	0.141
Side chain planarity	0.240	0.250
Inside/Outside distribution	1.012	1.000

Table S2. Griseofulvin induced nuclear fragmentation and nuclear accumulation of p53 in MCF-7 cells after 24 h.

Treatment	% Mono nucleated cells	% cells with fragmented nuclei	% Total cells which are p53 positive	% Mono-nucleated cells which are p53 positive	% p53 positive cells with fragmented nuclei
Control	98 ± 1.2	2 ± 1.2	5 ± 3	4 ± 2	26 ± 5
GF 15 μM	88 ± 2.6	12 ± 2.6	19 ± 2.4	11 ± 1	63 ± 3
GF 30 μM	77 ± 7.4	23 ± 7.4	29 ± 2.4	17 ± 7	67 ± 8
GF 60 μM	71 ± 8	29 ± 8	27 ± 4	11± 5	67 ± 10
GF 90 μM	69 ± 7	31 ± 7	25 ± 6	6 ± 1.2	72 ± 4

Data are average ± SD; approximately 600 interphase cells were scored in each concentration

Table S3. Griseofulvin induced nuclear fragmentation and nuclear accumulation of p53 in MCF-7 cells after 48 h.

Treatment	% Mono nucleated cells	% cells with fragmented nuclei	% Total cells which are p53 positive	% Mono-nucleated cells which are p53 positive	% p53 positive cells with fragmented nuclei
Control	99 ± 0.7	1 ± 0.7	3 ± 0.3	2 ± 0.5	23 ± 3
GF 15 μM	86 ± 10	14 ± 10	39 ± 13	31 ± 12	78 ± 12
GF 30 μM	67 ± 12	37 ± 12	30 ± 6	15 ± 6	58 ± 5
GF 60 μM	57 ± 9	43 ± 9	35 ± 6	13 ± 5	66 ± 5
GF 90 μM	50 ± 12	50 ± 12	37 ± 12	13 ± 9	61 ± 8

Data are average ± SD; approximately 600 interphase cells were scored in each concentration

Table S4. Griseofulvin induced nuclear fragmentation and nuclear accumulation of GFP-p53 in MCF-7 cells after 48 h.

Treatment	% Mono nucleated cells	% cells with fragmented nuclei	% Total cells which are p53 positive	% Mono-nucleated cells which are p53 positive	% p53 positive cells with fragmented nuclei
Control	98	2	3	2	1
GF 15 μM	70	30	35	12	42
GF 30 μM	51	49	41	12	46
GF 60 μM	41	59	50	17	58
GF 90 μM	53	37	42	11	47

Approximately 600 interphase cells were scored in each concentration

Table S5. Distance (Å) of griseofulvin (O1) with GTP, GDP and different drugs

	GTP (O4′)	GDP (O4′)	Colchicine (C11)	Vinblastine (C14)	Taxol (C43)
Site A	13.73	43.86	17.26	53.60	28.80
Site B	30.93	22.50	21.03	28.00	2.65
